# Transposon Invasion of the *Paramecium* Germline Genome Countered by a Domesticated PiggyBac Transposase and the NHEJ Pathway

**DOI:** 10.1155/2012/436196

**Published:** 2012-07-22

**Authors:** Emeline Dubois, Julien Bischerour, Antoine Marmignon, Nathalie Mathy, Vinciane Régnier, Mireille Bétermier

**Affiliations:** ^1^CNRS, Centre de Génétique Moléculaire, UPR3404, 1 Avenue de la Terrasse, 91198 Gif-sur-Yvette Cedex, France; ^2^CNRS, Centre de Recherches de Gif-sur-Yvette, FRC3115, 91198 Gif-sur-Yvette Cedex, France; ^3^Université Paris-Sud, Département de Biologie, 91405 Orsay, France; ^4^Université Paris Diderot, Sorbonne Paris Cité, Sciences du Vivant, 75205 Paris Cedex 13, France

## Abstract

Sequences related to transposons constitute a large fraction of extant genomes, but insertions within coding sequences have generally not been tolerated during evolution. Thanks to their unique nuclear dimorphism and to their original mechanism of programmed DNA elimination from their somatic nucleus (macronucleus), ciliates are emerging model organisms for the study of the impact of transposable elements on genomes. The germline genome of the ciliate *Paramecium*, located in its micronucleus, contains thousands of short intervening sequences, the IESs, which interrupt 47% of genes. Recent data provided support to the hypothesis that an evolutionary link exists between *Paramecium* IESs and *Tc1/mariner* transposons. During development of the macronucleus, IESs are excised precisely thanks to the coordinated action of PiggyMac, a domesticated *piggyBac* transposase, and of the NHEJ double-strand break repair pathway. A PiggyMac homolog is also required for developmentally programmed DNA elimination in another ciliate, *Tetrahymena*. Here, we present an overview of the life cycle of these unicellular eukaryotes and of the developmentally programmed genome rearrangements that take place at each sexual cycle. We discuss how ancient domestication of a *piggyBac* transposase might have allowed *Tc1/mariner* elements to spread throughout the germline genome of *Paramecium*, without strong counterselection against insertion within genes.

## 1. Introduction

Since the initial evidence for the existence of transposable elements (TEs) reported by McClintock [1], large-scale sequencing of the genome of a wide range of living organisms has highlighted the abundance of TE-derived sequences relative to the coding portion of genomes. Transposable elements, often considered as “selfish” or “parasitic” DNA, are mobile genetic elements that encode their own mobility enzymes and move from one genomic locus to another. Based on their transposition mechanisms, they can be classified into two main categories [2, 3]: class I elements transpose *via* the reverse transcription of an RNA molecule, while class II elements transpose *via* a DNA intermediate. Class II transposons, also called DNA transposons, are found in variable proportions among eukaryotic and prokaryotic genomes; for instance, they constitute the major fraction of resident TEs in bacteria (reviewed in [4]) but are underrepresented relative to class I elements in the human genome [5] and absent from the genome of the yeast *Saccharomyces cerevisiae* [6]. Among DNA transposons, cut-and-paste transposons move in two steps: (i) excision from the donor site, as a result of transposase-induced DNA cleavages at their ends and (ii) integration into the target site through strand transfer of their free 3′OH ends. The most widespread cut-and-paste transposons, which are found in eukaryotes and in prokaryotes, encode a so-called “DDE transposase,” an enzyme that bears a conserved triad of acidic residues (DDE or DDD) that catalyzes the excision and integration steps [2, 7]. Upon integration, these particular cut-and-paste transposons duplicate a short (2 to 15 bp) target sequence (TSD or target site duplication) on each side of the newly integrated copy (reviewed in [8]). Upon excision, staggered double-strand cleavages at their ends generally leave the two copies of the TSD at the donor site and the resulting double-strand break can be repaired by end joining [9–11]. This generates a “footprint” at the excision site, formed by two copies of the TSD flanking a few remaining bp from the transposon. However, if transposition takes places during replication, homologous recombination with the sister chromatid may restore the initial transposon copy at the donor locus, which leads to a net increase in transposon copy number in the genome [11–13].

Increase in transposon copy number may have detrimental effects on host fitness, since insertions can disrupt coding regions, modify the expression of adjacent cellular genes, or trigger ectopic recombination between distant transposon copies (reviewed in [8]). Several defense strategies have been developed by the host to inactivate transposition. In eukaryotes, posttranscriptional inactivation of TEs is mediated by homologous small RNAs, which may also induce histone and DNA methylation to inactivate the transcription of transposon genes (reviewed in [14]). In some hosts, like filamentous fungi, heavy mutagenesis of repeated sequences, a phenomenon named repeat-induced point mutation (or RIP), was also reported [15], but the involvement of small RNAs in this process has not been clearly demonstrated. As a result of these defense responses, many extant genomes harbor large numbers of defective copies of transposable elements that have lost their ability to transpose. Transposons, however, may also provide novel and advantageous functions to the host, as illustrated by the growing list of entire or truncated transposase genes found in eukaryotic genomes, either isolated or fused to genes encoding unrelated protein domains, which still appear to be expressed and encode proteins, but are not embedded within a mobile element anymore [16]. According to several criteria described in [8, 17], these genes have been domesticated to become cellular genes, but, in most cases, their function has not been elucidated. Most often, only putative transposase domains involved in nucleic acid binding have been conserved and may play a role in cellular DNA or RNA metabolism. Intriguingly, most domesticated transposases have lost their characteristic DDE (or DDD) signature and only for very few of them have evidence been obtained for *in vivo* or *in vitro* DNA cleavage activity. Remarkable examples of catalytically active domesticated DDE transposases were reported in different organisms: the RAG1 endonuclease, related to *Transib* transposases [18], catalyzes V(D)J recombination of immunoglobulin genes during the differentiation of lymphocytes in vertebrates (reviewed in [19]); alpha3, a domesticated *mutator*-like transposase, is involved in mating-type switching in the yeast *Kluyveromyces lactis* [20]; finally, SETMAR, identified in the human genome, carries a histone methyltransferase domain fused to the partially active catalytic site of a *mariner* transposase [21] and is thought to participate in the repair of DNA double-strand breaks, in the restart of stalled DNA replication forks and in chromosome decatenation ([22], reviewed in [23]).

Evidence for a role of domesticated *piggyBac* transposases in programmed genome rearrangements was reported recently in ciliates [24, 25]. Thanks to their unique nuclear dimorphism and to their original mechanism of programmed DNA elimination from their somatic nucleus, ciliates, and most specifically *Paramecium*, have emerged as novel model organisms for the study of the impact of transposable elements on genomes (for recent reviews, see [26, 27]). Here, we will present an overview of the life cycle of these unicellular eukaryotes and of the massive and developmentally programmed genome rearrangements that take place at each sexual cycle. We will discuss how the domestication of an ancient *piggyBac* cut-and-paste transposase in *Paramecium* might subsequently have allowed *Tc1*/*mariner* elements to spread throughout the germline genome, without strong counterselection against insertion within genes.

## 2. Developmentally Programmed Elimination of Germline Transposons and Related Sequences in Ciliates

### 2.1. Nuclear Dimorphism in Ciliates

**Figure 1:**
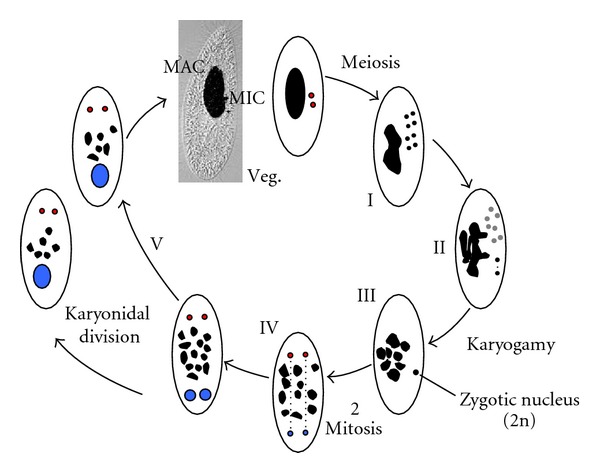
Nuclear dimorphism and the sexual cycle in *Paramecium*. The merged picture on top shows a vegetative *Paramecium* cell (Veg.), with its DAPI-stained nuclei (in black). The figure represents the major steps of the sexual cycle observed during autogamy, a self-fertilization process triggered by starvation. Upon starvation, the two germline diploid MICs (red) undergo meiosis to give rise to eight haploid nuclei (I), a single of which migrates to a specialized cell compartment, where it divides once to give two identical gametic nuclei (II). Meanwhile, the remaining seven meiotic products are degraded (grey dots in II) and the old MAC (black) gets fragmented into ~30 pieces. During karyogamy, two gametic nuclei fuse to form the diploid zygotic nucleus (III). The zygotic nucleus then undergoes two successive mitotic divisions (IV): after the second division, the nuclei which migrate to the anterior cellular pole become the new MICs of the sexual progeny (red), while those that localize to the posterior pole differentiate into new developing MACs (blue) and undergo programmed genome rearrangements. At the first cell division (or karyonidal division), the new MICs divide by mitosis and each of the two developing new MACs segregates into a daughter cell (V), where it continues to amplify the rearranged somatic genome to a final ploidy of ~800n. During conjugation (not shown), meiosis is triggered by the mating of two compatible sexual partners, which undergo reciprocal exchange of their haploid gametic nuclei. As a result, the zygotic nucleus in each partner is formed by the fusion of a resident and a migratory haploid nucleus. Exconjugants separate between the first and second divisions of the zygotic nucleus, and MAC development takes place as described for autogamous cells.

Ciliates form a deeply branching monophyletic group in the eukaryote tree [28]. These unicellular organisms are characterized by the coexistence, in their cytoplasm, of two functionally distinct types of nuclei (Figure 1). The diploid germline micronucleus (MIC) is transcriptionally silent during vegetative growth, but harbors the genetic information that is transmitted to the next sexual generation. According to ciliate species, the number of MICs per cell may vary (one in *Tetrahymena thermophila* and two in *Paramecium tetraurelia*, e.g.). Gene expression is carried out from the highly polyploid somatic macronucleus (MAC: ~800n in *Paramecium*), which is therefore essential for cell survival at all stages. During sexual events (conjugation between compatible mating types or, for some species, self-fertilization also called autogamy), MIC meiosis leads to the formation of haploid nuclei, one of which divides once to yield two identical gametic nuclei. The fusion of two gametic nuclei (reciprocally exchanged between mating partners during conjugation or originating from the same cell during autogamy) gives rise to the zygotic nucleus. In the meantime, the MAC is progressively degraded and is ultimately lost. New MICs and MACs differentiate from mitotic copies of the zygotic nucleus. Throughout this developmental process, the old MAC ensures all gene transcription and is progressively replaced by the new MAC [29]. Therefore, the development of a functional new MAC is essential for the survival of sexual progeny, once the old MAC has disappeared from the cell.

### 2.2. MIC and MAC Genomes Have Different Structures

MAC chromosomes of ciliates are shorter than their MIC chromosomes and apparently do not carry centromeres, which is consistent with the observation that the MAC divides through an amitotic process, with no chromosome condensation (reviewed in [30]). In contrast, the MIC undergoes mitosis and meiosis. Moreover, early studies of the complexity of MIC and MAC genomes pointed out that the two nuclei do not harbor the same DNA content, although they derive from the same zygotic nucleus. Indeed, the MIC genome contains additional sequences that are removed from the somatic genome during MAC development (reviewed in [31, 32]).

Pulse field electrophoresis analyses indicated that the size of* Paramecium *MAC chromosomes varies between 50 kb and 1 Mb [33]. The study of particular MAC chromosome ends in *P. primaurelia* [34] and *P. tetraurelia* [35, 36] revealed that they are capped by a mixture of G_3_T_3_ or G_4_T_2_ telomeric repeats added at heterogeneous positions by a single, error-prone telomerase [37, 38]; several telomere-addition regions distant of several kbp have been identified for some MAC chromosomes, each one extending over ~1 kb. The MAC genome sequence of *Paramecium tetraurelia* was obtained in 2006 by a consortium of European labs [39]. This study provided a global view of the structure of some 150 acentromeric MAC chromosomes and highlighted the fact that the somatic genome is streamlined for gene expression, with a very high gene density (78% coding) and essentially no repeated sequences. Even more striking, ~40,000 genes were annotated in the MAC genome, as a consequence of at least three successive whole genome duplications (WGD) during evolution of the *Paramecium aurelia* group of sibling species, to which *P. tetraurelia* belongs.

**Figure 2:**
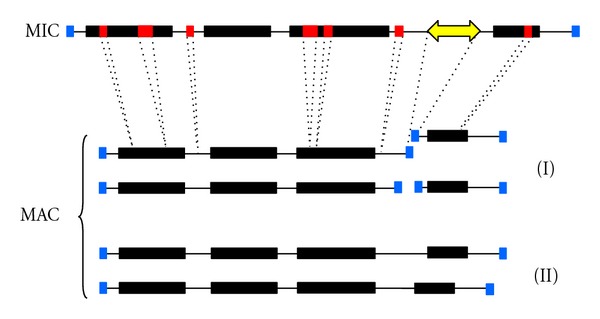
Schematic representation of the structure of the MIC and MAC genomes in *Paramecium*. On the MIC chromosome displayed on top, genes (black boxes) are interrupted by short internal eliminated sequences (IESs, in red), some of which are also found in noncoding regions (thin line). Repeated germline sequences (transposons, minisatellites, etc.) are symbolized by a yellow double-headed arrow. During MAC development, each MIC chromosome is amplified ~400-fold and gives rise to a population of heterogeneous MAC chromosomes. Indeed, imprecise elimination of repeated DNA is associated with alternative rearrangements: (I) chromosome fragmentation is observed and telomeres (blue squares) are added to new MAC chromosome ends; (II) the two chromosome arms that flank the eliminated germline region can be joined in an imprecise manner to generate internal deletions of heterogeneous sizes.

In contrast to somatic “chromosomes,” only limited knowledge of the number and structure of *Paramecium* germline chromosomes is available (Figure 2). Early microscopy studies proposed that 35 to 50 pairs of 1 to 7 Mb chromosomes harbor the genetic content of the MIC in *P. tetraurelia* [40]. Molecular analyses of a couple of germline regions encompassing MAC chromosome ends revealed no conserved nucleotide sequence motif for chromosome fragmentation in *Paramecium* [35]. This situation is quite different from that observed in other ciliates, in which consensus chromosome breakage sequences (CBS) were found at fragmentation sites (reviewed in [31]). Instead, the fragmentation of *Paramecium* MAC chromosomes seems to be associated with heterogeneous elimination of repeated germline sequences (minisatellites, germline transposons, etc.) located downstream of telomere addition sites [34, 41]. Southern blot hybridization experiments confirmed that known germline transposons are eliminated from the somatic genome during MAC development (O. Garnier, unpublished and [24, 42]). On a genome-wide scale, more work is clearly needed to gain full insight into the DNA content (and more specifically their TE landscape) of large germline regions that are eliminated from the MAC in association with chromosome fragmentation. In contrast, along chromosomes, the availability of a *λ* phage library of *P. tetraurelia* MIC DNA constructed by Preer et al. in 1992—which has represented a technical *tour de force* [43]—made it possible to compare the nucleotide sequence of particular MAC and collinear MIC loci; these studies led to the identification of short, noncoding sequences called IESs (internal eliminated sequences) that interrupt both coding and noncoding regions in the germline genome and are excised precisely from MAC chromosomes ([44], reviewed in [45, 46]). Recent genome-wide sequencing of a set of 45,000 IESs confirmed the early description of these sequences [41]. *Paramecium* IESs are very short (93% are shorter than 150 bp long and one-third are within the 26–30 bp size range) and each one appears to be single copy in the genome. Their exquisitely precise excision is essential for the recovery of a functional new MAC, since 47% of genes are interrupted by at least one of these intervening sequences in the germline genome [41]. Their only absolutely conserved feature is the presence of one flanking 5′-TA-3′ at each end, while a single TA is retained at their excision site on mature MAC chromosomes. Because of this conservation, *Paramecium* IESs have defined the family of the so-called “TA-IESs” [47]. In other ciliates, IESs are also eliminated during MAC development, but their structure varies from one species to the other. The existence of short TA-IESs has been reported in *Euplotes crassus* and *Oxytricha fallax*, while IESs in *Tetrahymena thermophila* are larger and are generally not flanked by TA repeats (reviewed in [31]).

### 2.3. Paramecium IESs Are Likely Remnants of *Tc1/mariner* Transposons

Statistical analysis of the nucleotide sequence of the ends of ~20 IESs from different *Paramecium aurelia* species was performed by Klobutcher and Herrick, who identified a degenerate 8 bp consensus (5′**TA**(C/T)AG(C/T)N(A/G)3′) that defines a loosely conserved terminal inverted repeat (TIR) at IES ends [48]. This consensus sequence, which includes the flanking TA, was confirmed by all the following analyses of increasing numbers of IESs [41, 46, 49]. Interestingly, it also matches the ends of the short TA-IESs found in *Euplotes *and *Oxytricha *[47] and also of *Tc1*/*mariner*-related transposons Tec1 and Tec2 present in high copy numbers in the germline genome of *Euplotes crassus *[48]. Based on the observation that TA-IESs and Tec transposons coexist in *Euplotes*, Klobutcher and Herrick proposed their IBAF model (invasion/bloom/abdicate/fade), according to which TA-IESs within a given ciliate species have evolved from *Tc1*/*mariner* transposons, which would have invaded the MIC genome and accumulated internal substitutions/deletions during evolution. Therefore, transposon remnants would have lost their coding capacity while being kept under strong selection pressure for their elimination from the somatic genome [50]. Interestingly, a common feature of *Tc1*/*mariner* transposons is their preference for TA dinucleotides as integration targets, which they duplicate upon insertion; thus, the conserved TAs at the boundaries of TA-IESs would simply be the TSDs generated by integration of ancestral *Tc1*/*mariner*-related TEs.

**Figure 3:**
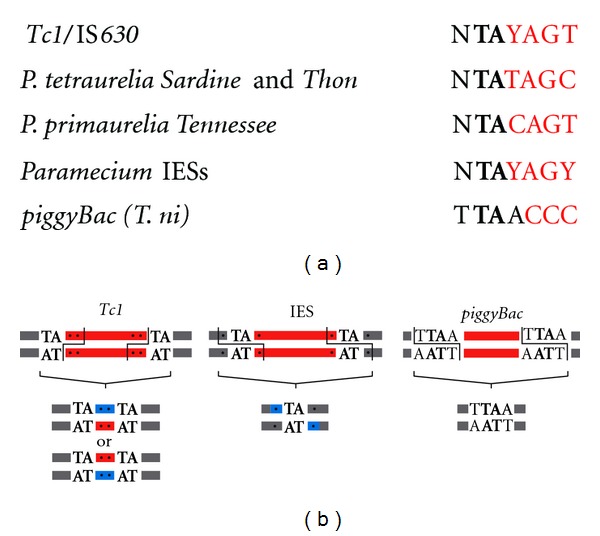
*Paramecium* IES excision: a comparison with *Tc1*/*mariner* and *piggyBac* transposons. (a) Nucleotide sequence alignment of the ends of *Paramecium* IESs with the termini of *Tc1* transposons (general *Tc1/*IS*630 *consensus and transposon families identified in *Paramecium*) and of the *piggyBac* element from *Trichoplusia ni*. Flanking sequences are in black (the conserved TA found at each boundary is highlighted in bold) and internal nucleotides are in red. Note that for *piggyBac*, the target site duplication is made of 4 base pairs (TTAA in black). (b) The geometry of double-strand DNA cleavages introduced by *Tc1* (left) and *piggyBac* (right) transposases is shown on top, together with that of PiggyMac-dependent DSBs detected at *Paramecium* IES ends (middle). The conserved TAs are represented by black bold letters. Based on their transposition mechanism, *Tc1* and *piggyBac* transposons are delimited by their cleaved 3′ ends and are represented by red lines. By analogy with *Tc1*, IESs are drawn as red lines bounded by two flanking TAs (in black), although this does not reflect the actual position of DNA cleavages. At the bottom of each panel, the structure of chromosomal junctions formed after excision from the donor site is shown. For *Tc1* transposons and *Paramecium* IESs, the nucleotides that are neosynthesized during gap filling and repair are represented in blue.

The IBAF model for the evolutionary origin of IESs has recently obtained further support in *Paramecium*. Transposon-like sequences were indeed identified in the heterogeneously eliminated fraction of the germline genome of different *P. aurelia *strains [34, 41]. Sequence alignment of these elements has led to the establishment of a consensus for each family (*Tennessee* in *P. primaurelia*, *Sardine* and *Thon* in *P. tetraurelia*) and to the unambiguous identification of open reading frames encoding putative transposases harboring the characteristic DD(35)E triad of *Tc1*/*mariner/*IS*630* transposons. Reminiscent of the situation described in* E. crassus*, the six outward terminal nucleotides (including the flanking TA) of the long (500 to 700 bp) and complex TIRs of the *Sardine* and *Thon* elements of *P. tetraurelia* match the consensus of IES ends (Figure 3(a)). Analysis of the three WGDs that took place during the evolutionary history of *P. tetraurelia* provided evidence that IESs have appeared continuously in the germline genome and that their size tends to shorten over time [41]. Among the largest IESs (>500 bp), a few closely related IESs are inserted at nonhomologous germline loci. Some of them exhibit significant sequence similarities with the long TIRs of known TEs from the *Thon* family and are excised from MAC chromosomes just like any other IES. The existence of these “solo TIRs” provides further support to the notion that some IESs at least have derived from recently mobile TEs from the *Tc1/mariner* family. Genetic evidence indicates that the conserved TAs, which are supposed to represent the TSD created by integration of the ancestral *Tc1*/*mariner*, are essential for the developmentally programmed excision of IESs [51–55].

## 3. IES Excision in **Paramecium **: A Cut-and-Close Reaction Mediated by a Domesticated Transposase

### 3.1. IES Excision Is Related to Cut-and-Paste Transposition of piggyBac, Not of *Tc1/mariner* Elements

One of the assumptions of the IBAF model is that, at first, ancestral invading germline *Tc1*/*mariner* transposons were eliminated from the somatic genome during MAC development, thanks to the action of their own transposase [50]; thus, programmed genome rearrangements have allowed TEs to proliferate in the MIC, with little or no effect on the phenotype of the cell, as long as they are correctly excised from the MAC. Then, at some point during evolution, a cellular gene (possibly a domesticated or preexisting *Tc1*/*mariner* transposase gene) took over the catalysis of excision of all these elements, allowing them to accumulate internal mutations and give rise to current IESs, while still being able to excise from the MAC. As already discussed [45], one caveat of this model is that *Tc1*/*mariner* transposition leaves a characteristic footprint at the excision (or donor) site [10], while IESs are excised precisely at the nucleotide level, leaving only one copy of the original duplicated TA at the excision junction (Figure 3(b)).

**Figure 4:**
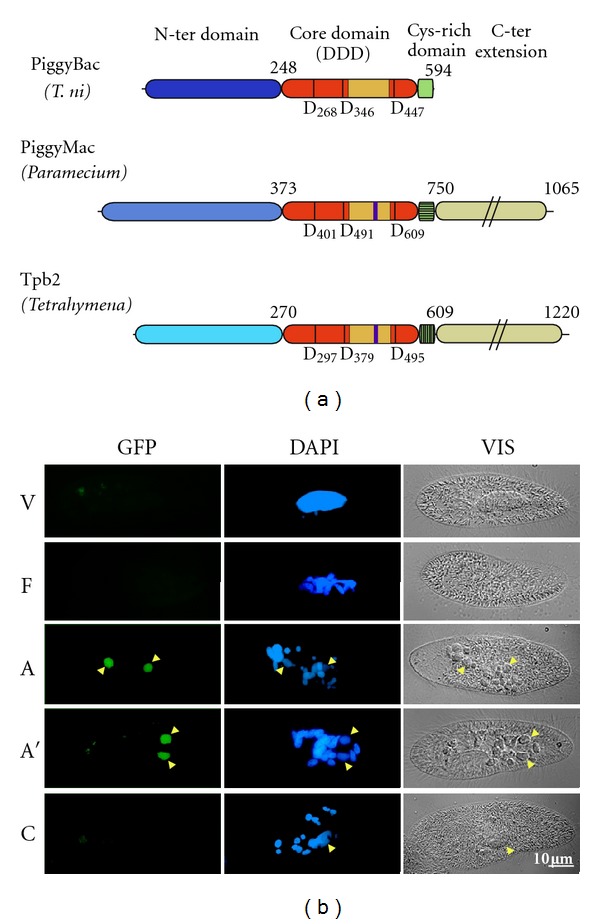
PiggyMac: a domesticated PiggyBac transposase in *Paramecium*. (a) Conserved domains in ciliate domesticated transposases. PiggyMac and the related Tpb2 protein from *Tetrahymena thermophila* were aligned with the transposase of the *piggyBac* transposon isolated from *Trichoplusia ni*, using the MUSCLE software (http://www.ebi.ac.uk/Tools/msa/muscle/). The conserved catalytic core domain is represented in orange, and the putative DDD catalytic residues are indicated for each protein (numbers refer to amino acid positions in the primary sequence). A *β* strand-rich module (yellow box) can be predicted between the second and third catalytic residues using the PSIPRED package (http://bioinf.cs.ucl.ac.uk/psipred/); in ciliate proteins, additional residues inserted within this module are represented by a purple bar. The cysteine-rich domain is drawn as a light green box at the C-terminus of PiggyBac, and corresponding variant domains in Pgm and Tpb2 as hatched boxes. The C-terminal coiled-coil extensions of ciliate domesticated proteins are not drawn to scale (light beige boxes). The divergent N-terminal domains of the three proteins are represented in different shades of blue. (b) A PiggyMac-GFP fusion localizes to developing new MACs. A transgene encoding a C-terminal GFP fusion expressed under the control of endogenous *PGM* transcription signals was microinjected into the MAC of vegetative cells (Dubois, unpublished). During autogamy, cells were fixed and nuclei were stained with DAPI and observed with a Zeiss epifluorescence microscope (magnification 630x). No GFP fluorescence was observed in vegetative cells (V) or at early stages during autogamy, when the old MAC starts its fragmentation (F). The GFP fusion protein was detected specifically in the two developing MACs of autogamous cells (arrowheads in A and A′) and GFP fluorescence disappeared from the new MAC after karyonidal division (C).

Molecular analyses of IES excision intermediates formed *in vivo* during sexual processes in *P. tetraurelia* provided important information about the mechanisms involved in this process. IES excision starts after a few rounds of endoduplication of the germline genome have taken place, so that at least 16 copies of each IES need to be excised in each developing MAC [56]. It is initiated by 4 bp staggered double-strand DNA cleavages at both ends of each IES, centered on the conserved TA dinucleotides [57]. As a result, transient double-strand breaks (DSBs) with characteristic 4-base 5′ overhangs can be detected by ligation-mediated PCR during MAC development, at the ends of linear excised IES molecules and at flanking MAC-destined DNA ends (Figure 3(b) and [57, 58]). Strikingly, these DSBs have the same geometry as those catalyzed *in vitro* by *piggyBac* transposases [59]. Indeed, *piggyBac* cut-and-paste transposons duplicate a 5′-TTAA-3′ target site upon integration, and when they transpose to a new locus, their transposase cleaves DNA on each side of each duplicated TSD to generate a 5′ TTAA overhang [59]. Thus, *piggyBac* excision is highly precise and reconstitutes the TTAA sequence at the donor site [60]. The discovery of *PiggyMac* (*PGM*), a domesticated *piggyBac* transposase gene in *P. tetraurelia*, represented a significant breakthrough towards the identification of protein partners involved in IES excision [24]. This gene is only expressed during sexual processes, with an induction peak during the development of new MACs, which corresponds to the time when IES excision starts. It encodes a large 1065 aa protein with a recognizable central domain homologous to the transposase of *piggyBac* transposons, including a potentially active DDD catalytic triad (Figure 4(a)). During sexual processes, Pgm-GFP fusion proteins were found to localize specifically in the developing new MACs, in which IES excision takes place (Figure 4(b) and [24], Dubois, unpublished). In cells silenced for expression of the *PGM* gene, IES excision is blocked as well as other known programmed genome rearrangements (chromosome fragmentation and heterogeneous elimination of *Sardine* transposons); as a result, strong lethality is observed in the sexual progeny. Nuclei of *PGM*-silenced cells (purified during the development of new MACs, before the cells die) provided the source of DNA that was used for whole-genome sequencing and identification of the set of 45,000 IESs described above [41]. Indicative of a catalytic function of Pgm, microinjection of a mutant transgene encoding a protein in which the DDD catalytic triad was switched to AAA induces a dominant negative effect on the survival of sexual progeny, while a normal phenotype is obtained with a wild-type transgene (Dubois, unpublished). Thus, even though *Paramecium* IESs are probably relics of *Tc1*/*mariner* transposons, their precise excision from the MAC genome appears to be carried out by a domesticated transposase related to a different family of transposable elements, the *piggyBac* family.

DNA transposons generally assemble a synaptic nucleoprotein complex called the “transpososome,” which includes both transposon ends and oligomers of the transposase (see [61] for a review). Assembly of this complex activates the successive hydrolysis and transesterification steps that ultimately lead to transposon excision. Likewise, genetic evidence has indicated that IES excision in *Paramecium* involves an interaction between the two ends of each IES, before DNA cleavage [62]. For three IESs of different sizes (28, 66, and 370 bp), it was indeed shown that a mutation within the TA at one end not only inhibits cleavage of the mutant end but also strongly impairs DNA cleavage at the wild-type end of the same IES. Moreover, thorough analysis of the currently available set of 45,000 IESs of *P. tetraurelia* provided support to the hypothesis that IES excision, similar to transposition, involves the formation of an intramolecular DNA loop on a double-strand substrate [41]. Indeed, the size distribution of IESs exhibits a striking 10 bp periodicity, which coincides with the length covered by one turn of the DNA double helix. This suggests that interactions between Pgm molecules bound at each end of an IES depend critically on helical phasing, especially for very short sequences (93% of *Paramecium* IESs are shorter than 150 bp, the persistence length of double-strand DNA). As discussed for other systems (site-specific recombination, transposition, or repression), DNA looping between very closely spaced sites might also be favored by DNA bending factors and/or local melting of the double helix [63].

### 3.2. How May PiggyMac Recognize Paramecium IESs?

The ends of cut-and-paste transposons are generally made of two parts: an internal sequence-specific binding site for their cognate transposase and a few nucleotides at their termini that constitute the DNA cleavage site *per se* (see, for example, [64]). For *piggyBac*, the site cleaved by the transposase is the TTAA duplicated target sequence on each side of the integrated copy of the element. A 13 bp terminal repeat (TR) and a 19 bp internal repeat (IR) separated by a spacer are present in inverted orientation at each end of the element and may be binding sites for the transposase [65, 66]. Analysis of the nucleotide sequence of 45,000 IESs from *P. tetraurelia* showed that the TTAA tetranucleotide is actually largely underrepresented at IES ends (Marmignon, unpublished), even though the sequence cleaved by PiggyMac at the termini of *Paramecium* IESs bears some similarity with the site cleaved by the PiggyBac transposase (i.e., a 4 bp sequence with a central TA). Furthermore, neither TR nor IR repeats are found at the ends of *Paramecium* IESs. The situation is even more striking for *Tetrahymena* IESs, which depend on a close PiggyMac homolog, the PiggyBac-like transposase called Tpb2, for their elimination [25]; indeed, the sequence cleaved at their ends (5′ANNNNT3′) does not even carry a conserved central TA [67]. This suggests that ciliate domesticated *piggyBac* transposases do not recognize a specific nucleotide motif at IES ends and raises the question of how germline sequences are targeted for elimination.

Part of the answer may lie in the epigenetic mechanisms that control programmed genome rearrangements in *Paramecium* and *Tetrahymena* (reviewed in [26, 27, 69, 70]). It was proposed for both ciliates that a comparison between the DNA content of parental MIC and MAC genomes takes place during MIC meiosis through the annealing of two kinds of noncoding RNA molecules. Short RNAs, also called scnRNAs (25 nt in *Paramecium*, 28 nt in *Tetrahymena*), are generated by a specialized RNA interference pathway from noncoding RNA precursors transcribed specifically from the MIC during meiosis. According to the “scanning” model, these scnRNAs would pair to larger transcripts that are produced constitutively by generalized transcription of the parental MAC genome, which was rearranged during the previous sexual cycle. Those scnRNAs that do not find homologous MAC sequences, and therefore represent the fraction of the germline genome that was absent from the parental MAC, are then imported into the new developing MAC, in which they are thought to target the deletion of homologous sequences. In *Tetrahymena*, the methylation of IES-associated histones is clearly one of the scnRNA-dependent epigenetic modifications that trigger the elimination of heterochromatin regions [71, 72]. In contrast, the putative epigenetic marks that are deposited by scnRNAs on *Paramecium* germline eliminated sequences have not been identified yet, especially for IESs, the vast majority of which are much shorter than the length of DNA wrapped around a nucleosome (~150 bp). Whatever the exact mechanism may be, a strong implication of the scanning model is that ciliates tend to reproduce their pattern of developmentally programmed genome rearrangements from one sexual generation to the next. Thus, epigenetic control may have contributed to loosen the requirement for a specific nucleotide sequence to direct ciliate domesticated PiggyBac transposases towards regions that have to be eliminated from the developing MAC. Quite interestingly, PiggyMac and Tpb2 present variant domains relative to PiggyBac transposases (within their catalytic site and a downstream cysteine-rich region) and have acquired long C-terminal extensions (Figure 4(a)); the role of these domains in IES recognition still has to be elucidated.

### 3.3. The Cellular Nonhomologous End Joining Double-Strand Break Repair Pathway Closes IES Excision Sites

**Figure 5:**
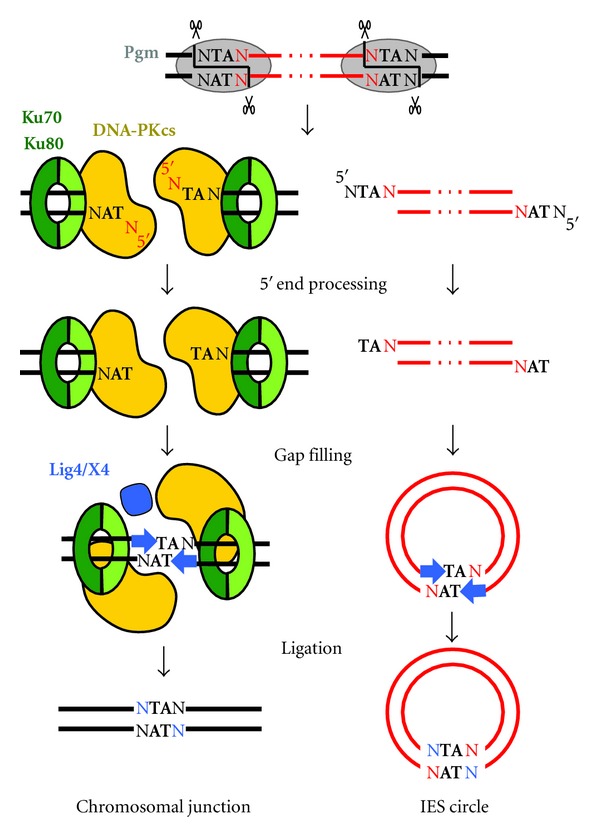
Molecular mechanism of IES excision in *Paramecium*. The successive DNA intermediates that are formed during IES excision are displayed, with IESs in red and flanking MAC-destined DNA in black. The proteins that were shown to be required for proper IES excision are also represented. The first step is the introduction of 4-base staggered double-strand breaks at each IES end and depends on the PiggyMac domesticated transposase (Pgm). According to available knowledge of the classical NHEJ pathway in other organisms, a Ku70/Ku80 dimer is proposed to bind to each broken flanking DNA end and recruits the DNA-PKcs catalytic subunit. The last steps of the reaction were proposed to take place within a paired-end intermediate guided by annealing of the central TA present on each 5′ overhang [57]. The proteins involved in the removal of the 5′ terminal nucleotide have not been identified. For the 3′ processing step, the ligase IV is required for recruiting or activating a gap-filling DNA polymerase, which adds one nucleotide to the recessive end, prior to final ligation. A similar mechanism is proposed for the circularization of excised linear IES molecules (right part of the figure), providing that they are long enough. IES circles do not replicate and are actively degraded.

Thanks to the particular cleavage properties of their transposase, the transposition of *piggyBac* transposons leaves no footprint at the donor site (Figure 3(b)), and excision junctions can be closed through direct annealing of the fully complementary 5′-TTAA-3′ overhangs generated on flanking DNA ends, with no need for any additional processing step [59]. For *Paramecium* IESs, however, the situation is quite different, since only the central TA is conserved on the 4-base overhangs created by Pgm-dependent cleavage. It was proposed that the closure of IES excision junctions on MAC chromosomes involves partial pairing of the flanking ends through annealing of their conserved TAs and limited additional processing (removal of the unpaired 5′-terminal nucleotides and gap-filling by addition of one nucleotide at each 3′ recessive end), before the final ligation step (Figure 5 and [57]). IESs are assumed to be excised as linear molecules and, at least for those larger than 200 bp, to be circularized in a second step using the same pathway (partial pairing of overhangs, 5′ and 3′ processing, ligation). The enzymes that carry out the additional processing steps have not been identified. However, recent work uncovered the essential role played by the ligaseIV and its partner Xrcc4 in the closure of IES excision sites and the circularization of excised IESs [58]. LigaseIV and Xrcc4 are core actors of the nonhomologous end joining (NHEJ) pathway, which repairs DSBs through the direct joining of broken ends, without requiring sequence homology [73]. Two very closely related *LIG4* genes originating from the most recent WGD and a single *XRCC4* gene were identified in the genome of *P.tetraurelia*. Their expression reaches a peak during MIC meiosis, even before new MACs have differentiated from mitotic copies of the zygotic nucleus; this implies that induction of DSB repair genes is part of a developmental program in *Paramecium* rather than a response to DNA damage. In cells depleted either for ligaseIV or Xrcc4, Pgm-dependent cleavages are introduced normally, but no detectable chromosomal—nor circular—junctions are formed; unrepaired DSBs accumulate at IES excision sites as well as linear forms of excised IESs. Noteworthy, DSBs are processed normally at their 5′ end in ligase IV-depleted cells (removal of the 5′terminal nucleotide), but no nucleotide addition is observed at their 3′ recessive end [58]. As already inferred from *in vitro* studies of reconstituted eukaryotic NHEJ systems [74], this indicates that the ligaseIV participates in the recruitment or activation of a gap-filling DNA polymerase, prior to end joining (Figure 5).

**Figure 6:**
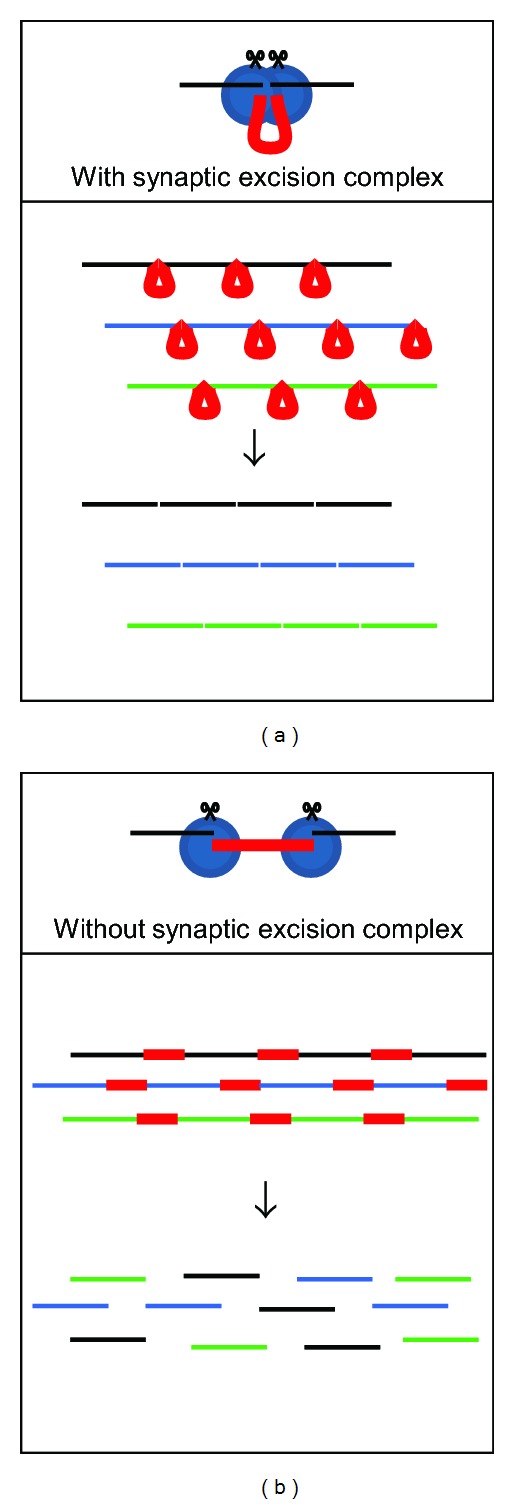
PiggyMac and the NHEJ pathway orchestrate accurate assembly of MAC chromosomes. On the diagrams shown on top of each panel, the IES is drawn in red and its flanking DNA in black. The excisase complex, which includes PiggyMac and putative additional partners, is represented in blue. In bottom panels, different germline chromosomes are displayed in different colors, with IESs in red. (a) If a synaptic complex is formed prior to cleavage, adjacent MAC-destined chromosome fragments are brought together, which might favor their alignment during the repair step, therefore limiting the risk of translocation. (b) In the absence of a synaptic complex, IES excision could lead to catastrophic chromosome breakage and translocation, as described in [68].

The participation of actors of the NHEJ pathway in the final step of IES excision raises the question of how accurate end joining is achieved following the massive introduction of programmed DSBs throughout the genome. Indeed, given the number of IESs per haploid genome, thousands of DSBs are introduced all along chromosomes within a restricted time window during MAC development. The formation of a Pgm-containing synaptic excision complex prior to IES end cleavage might contribute to hold together adjacent fragments of MAC-destined DNA, ensuring, therefore, that somatic chromosomes are assembled in the right order during DSB repair (Figure 6). Furthermore, the human Xrcc4 protein was recently shown to form filaments with another NHEJ factor, Cernunnos (or XLF), independently of ligase IV [75–77]. These filaments are thought to promote the bridging between broken DNA ends [78]. Two Cernunnos homologs are encoded by paralogs of the recent WGD in *P. tetraurelia*, and their expression is induced during sexual processes [58]. During IES excision, such filaments may provide an alignment scaffold and favor the correct assembly of broken MAC ends. At the nucleotide level, an additional requirement for assembly of a functional MAC genome is the highly precise joining of each IES excision site to reconstitute open reading frames. Increasing evidence that the “classical” NHEJ pathway is inherently precise ([79], reviewed in [80]) has pointed to the key role played by the Ku70/Ku80 heterodimer in protecting broken DNA ends against resection and inhibiting other DSB repair pathways, such as alternative end-joining (which would create imprecise deletions) or homologous recombination (which would restore the non-rearranged molecule). Ku proteins have been conserved through evolution, from bacteria to humans [81], and several genes encoding putative Ku70 and Ku80 homologs were found in the genome of *P. tetraurelia* [58]. Efficient recruitment of Ku proteins at IES excision sites probably plays a determinant role in the precision of DNA rearrangements. *P. tetraurelia* also harbors a unique gene encoding a homolog of the DNA-PKcs, a DNA-dependent protein kinase (Malinsky et al., in preparation) that interacts with the Ku dimer, facilitates the synapsis of broken DNA ends, and, after autophosphorylation, activates downstream NHEJ proteins [82]. The conservation of DNA-PKcs in *Paramecium*, even though this protein has been lost from other model organisms such as budding yeast or *Drosophila*, suggests that this protein was present in the ancestral eukaryotic NHEJ core machinery. Functional inactivation of the *KU* and *DNA-PKcs* genes by RNA interference indicates that Ku70/Ku80 and the DNA-PKcs homolog are required for IES excision (Marmignon, unpublished; Malinsky et al., in preparation). Strikingly, among the three *KU80* genes identified in the genome, only one is specifically expressed during MAC development and appears to have acquired a specialized function in genome rearrangements (Marmignon, unpublished).

### 3.4. Revisiting the IBAF Model

The availability of a genome-wide set of ~45,000 IESs in *Paramecium tetraurelia* has broadened our current view of the evolutionary history of the germline genome of ciliates [41]. All IESs that have been identified so far in *Paramecium* belong to the TA-IES family. Consistent with the IBAF model proposed by Klobutcher and Herrick, some of them at least seem to have evolved from ancestral *Tc1*/*mariner*-related transposons, still recognizable in the fraction of the MIC genome that is eliminated in an imprecise manner. This putative evolutionary link between IESs and TEs is similar to that proposed for *Euplotes*, a distant spirotrichous ciliate in which Tec transposons and related TA-IESs are excised precisely from the MAC genome, although the details of the mechanism may be somewhat different from IES excision in *Paramecium*. In particular, the enzyme responsible for IES excision in *Euplotes* has not been identified yet. In another stichotrichous ciliate, *Oxytricha*, at least three families of TBE transposons, also related to the *Tc1* family and initially designated as telomere-bearing elements, have been identified in the eliminated fraction of the MIC genome [83]. Along the lines of the IBAF model, RNA interference experiments have suggested that the TBE transposase itself mediates the elimination of TBE transposons from the somatic genome [84]. It appears to be also involved in other genome rearrangements reported in *Oxytricha*, such as IES excision and the unscrambling of a subset of genes, for which macronuclear-destined sequences are not collinear in the MIC and MAC genomes. This situation has provided a nice example of mutualism, rather than domestication, between resident transposons and their host (discussed in [85]).

**Figure 7:**
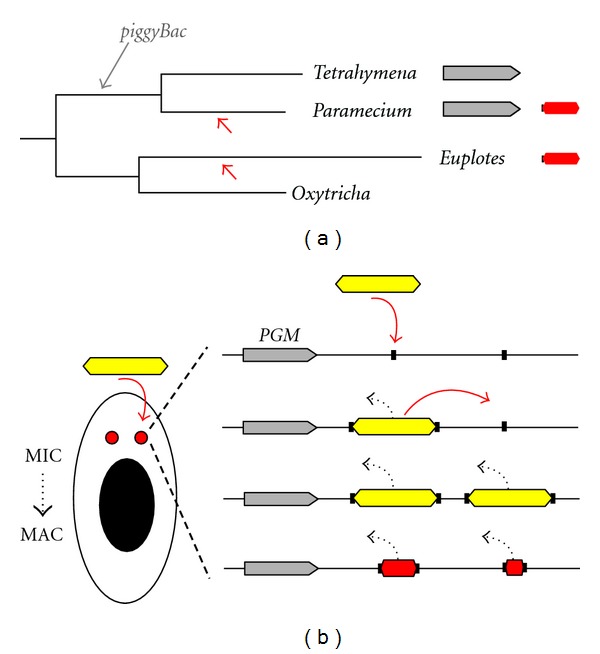
Evolutionary scenario for the origin of *Paramecium* IESs. (a) Putative timing for transposon invasion in the ciliate phylum (ciliate tree adapted from [28]). Identification of closely related domesticated *piggyBac* transposases in *Tetrahymena* and *Paramecium* (grey boxes) led to the hypothesis that a *piggyBac* transposon invaded the germline genome of one of their common ancestors (grey arrow), prior to the divergence between these two ciliate lineages. Because of the absence of TA-IESs in *Tetrahymena*, only the germline genome of *Paramecium* is thought to have undergone subsequent invasion by *Tc1*/*mariner* transposons (red arrowhead). TA-IESs (red box flanked by two black squares) and related transposons were found in the more distant ciliate *Euplotes*, but the protein(s) required for their developmentally programmed excision have not been identified. (b) In the revisited version of the IBAF model in *Paramecium*, the ancestor of the *PGM* gene (in grey) was already present when the first *Tc1*/*mariner* transposon (yellow box) started to invade the MIC. During the blooming step, Pgm may have been recruited to rid the genome from deleterious transposon insertions within genes. Thanks to the preexistence of the Pgm domesticated transposase and to the NHEJ repair pathway, *Tc1*-related transposons could be excised precisely from the somatic genome of the next sexual generation (programmed elimination from the MAC is represented by black dotted arrows), between the two duplicated copies of their TA target site (black squares). This has allowed invading *Tc1*/*mariners* to spread throughout the germline genome as a consequence of transposition catalyzed by their own transposase (mobility inside the MIC is symbolized by red arrows). During evolution, most copies of *Tc1/mariner* transposons have lost their coding capacity and have shortened in size, while being kept under selection pressure for their Pgm-dependent precise excision from the MAC, to give the currently known IESs (red boxes).

In *Paramecium*, the discovery that elimination of IESs and *Tc1*/*mariner*-like transposons depends on a domesticated transposase related to the *piggyBac* family has provided an unexpected extension of the IBAF hypothesis [24]. As discussed previously [24, 41], the existence of a catalytically active PiggyMac homolog, Tpb2, also required for programmed genome rearrangements in *Tetrahymena thermophila* [25], indicates that domestication of a PiggyBac transposase occurred early during ciliate evolution, before the divergence between *Paramecium* and *Tetrahymena* (Figure 7(a)). The initial role of this ancestral PiggyBac transposase might have been to cope with a first invasion of *piggyBac* elements, by removing them from ciliate genomes. It may then have been recruited to carry out the elimination of other unrelated germline sequences from the MAC genome. Intriguingly, except for a few TTAA-IESs that may originate from *piggyBac* transposons (some of which add 3′ exons to genes that would be expressed transiently during MAC development), *Tetrahymena* IESs are generally not flanked by TA dinucleotides and differ significantly from those of *Paramecium*; they are larger and are usually multicopy elements, their excision generates microheterogeneity at chromosomal junctions, and they are very rarely found within coding sequences [26, 86]. This suggests, therefore, that invasion of the *Paramecium* germline genome by *Tc1*/*mariner* transposons took place after the separation of the two ciliate lineages (Figure 7(a)). This idea has been supported by an analysis of IES evolution in *Paramecium*, which led to the conclusion that the majority of TA-IESs appeared between the intermediate and recent WGDs [41], that is after divergence of *Paramecium *and* Tetrahymena* [39]. In *Paramecium*, the ability of PiggyMac and the NHEJ pathway to carry out the precise excision of *Tc1*/*mariner*-related elements from the MAC may have allowed these transposons to spread throughout the germline genome without harmful consequences on gene expression (Figure 7(b)). Thus, thanks to nuclear dimorphism and to the existence of a precise mechanism for transposon elimination from the somatic genome, *Paramecium*, in contrast to other organisms, may have tolerated insertions within genes. This raises the question of whether currently known IESs are the relatively harmless remnants of ancient *Tc1*/*mariner* invaders or whether they have acquired some useful function for the cell. As suggested earlier [87], some of them may contribute to the structuration of MIC chromosomes, for example, by providing centromere-related functions or ensuring the condensation of chromosomes. IESs may also have a regulatory role, if they carry sequences that can control transient gene expression specifically before they are removed from genes during genome rearrangements.

## 4. Conclusion

A recent study of ~10 million genes annotated in sequenced genomes from individual bacteria, archaea, eukaryotes, and viruses as well as in metagenomes, has pointed to the remarkable evolutionary success of transposase genes, which appear to be “the most abundant, the most ubiquitous genes in nature” [88]. This brought further support to the idea that transposons should not simply be considered as selfish or parasitic elements, but also as a source of novel and sometimes essential functions for their host. In mammalian genomes for instance, numerous transposase genes seem to have been domesticated, but, for the most part, their function has remained elusive [8]. The best documented example is the RAG1 nuclease involved in V(D)J recombination, a process that generates the highly diverse repertoire of immunoglobulin genes in differentiating B and T lymphocytes (reviewed in [19]). RAG1 is clearly a domesticated transposase from the *Transib* family, and its target sites within immunoglobulin genes, also called the recombination signal sequences, present significant sequence similarities with the TIRs of *Transib* transposable elements [18]. As in *Paramecium*, the NHEJ double-strand break repair pathway has been recruited in this system to join the coding (and signal) ends and assemble functional immunoglobulin genes. In V(D)J recombination, however, additional factors (such as nucleases and a template-free DNA polymerase) contribute to the observed variability of the coding junctions.

In addition to being yet another example of a catalytically active domesticated transposase involved in programmed DNA elimination during differentiation, PiggyMac in *Paramecium* represents a novel variation on the theme of how a genome can cope with invasion by transposable elements. Here, a domesticated *piggyBac* transposase, the NHEJ pathway and epigenetic control by noncoding RNAs orchestrate a highly precise and accurate system for the programmed elimination of transposon-related sequences from somatic chromosomes. As discussed in [89], recent observations have indicated that some IESs in *Paramecium* may carry promoters or parts of coding sequences, the excision of which would be regulated during the development of a new MAC and could also be submitted to homology-dependent epigenetic control of the old MAC. Whether IES excision may have provided an additional layer of variability for the control of gene expression at the genome-wide scale is an attractive hypothesis that will need to be investigated.
